# Quantitative Multicomponent T2 Relaxation Showed Greater Sensitivity Than Flair Imaging to Detect Subtle Alterations at the Periphery of Lower Grade Gliomas

**DOI:** 10.3389/fonc.2021.651137

**Published:** 2021-03-22

**Authors:** Pietro Bontempi, Umberto Rozzanigo, Dante Amelio, Daniele Scartoni, Maurizio Amichetti, Paolo Farace

**Affiliations:** ^1^ Proton Therapy Unit, Hospital of Trento, Azienda Provinciale per i Servizi Sanitari (APSS), Trento, Italy; ^2^ Radiology Department, Hospital of Trento, Azienda Provinciale per i Servizi Sanitari (APSS), Trento, Italy

**Keywords:** magnetic resonance imaging, radiotherapy, glioma, multicomponent T2 analysis, myelin water imaging, relaxometry

## Abstract

**Purpose:**

To demonstrate that quantitative multicomponent T2 relaxation can be more sensitive than conventional FLAIR imaging for detecting cerebral tissue abnormalities.

**Methods:**

Six patients affected by lower-grade non-enhancing gliomas underwent T2 relaxation and FLAIR imaging before a radiation treatment by proton therapy (PT) and were examined at follow-up. The T2 decay signal obtained by a thirty-two-echo sequence was decomposed into three main components, attributing to each component a different T2 range: water trapped in the lipid bilayer membrane of myelin, intra/extracellular water and cerebrospinal fluid. The T2 quantitative map of the intra/extracellular water was compared with FLAIR images.

**Results:**

Before PT, in five patients a mismatch was observed between the intra/extracellular water T2 map and FLAIR images, with peri-tumoral areas of high T2 that typically extended outside the area of abnormal FLAIR hyper-intensity. Such mismatch regions evolved into two different types of patterns. The first type, observed in three patients, was a reduced extension of the abnormal regions on T2 map with respect to FLAIR images (T2 decrease pattern). The second type, observed in two patients, was the appearance of new areas of abnormal hyper-intensity on FLAIR images matching the anomalous T2 map extension (FLAIR increase pattern), that was considered as asymptomatic radiation induced damage.

**Conclusion:**

Our preliminarily results suggest that quantitative T2 mapping of the intra/extracellular water component was more sensitive than conventional FLAIR imaging to subtle cerebral tissue abnormalities, deserving to be further investigated in future clinical studies.

## Introduction

Non-invasive characterization of brain water can provide valuable insights for a better understanding of pathologic conditions affecting the central nervous system (CNS). Previous studies have used T2 relaxometry to investigate brain water (1), as the T2 distribution for normal brain *in vivo* can be represented by multiple components, corresponding to water in different compartments (2). A short T2 component (T2 around time = 10–50 ms) has been associated with water trapped between myelin bilayers, an intermediate component (T2 around 60–100 ms) has been linked to intra/extracellular water and a long T2 component has been associated with cerebrospinal fluid (CSF).

T2 relaxometry is at the basis of myelin water imaging (MWI) for the quantification of myelin component (3–5). Well-studied applications have been developed to investigate demyelinating diseases in the CNS, such as multiple sclerosis and neuromyelitis optica (6). However, to our knowledge only an attempt to apply multicomponent T2 relaxometry for the investigation of brain tumors has been performed so far (7).

In a prospective study that enrolled patients who received proton irradiation to the CNS, we investigated the potential of multicomponent T2 relaxometry in radiation oncology. Herein, we describe the method for acquisition and analysis of multicomponent T2 relaxometry and the first data obtained on a sample of six lower grade glioma patients. The data accidentally showed that decomposing T2 can be more sensitive than conventional FLAIR imaging for detecting subtle tissue alterations in the peri-tumoral region.

## Materials and Methods

### Subjects

Patients were recruited from an ongoing institutional review board approved study on the application of MWI in radiotherapy. Written informed consent was obtained for all participants. Six patients affected by lower grade glioma WHO grades II–III were selected. All the lesions had no or marginal enhancement on post-contrast imaging. Patients characteristics, dose prescriptions and time between surgery and treatment are listed in **Supplementary Table S1**. All the patients were irradiated by fractionated proton therapy (PT), uniformly delivered on the clinical target volume (CTV). FLAIR images were manually segmented to identify gross tumor volume (GTV) on pre-PT by two expertized radiation oncologists (DA and DS), using the contouring tools of the treatment planning system. For brainstem gliomas (patients #1 and #6, see **Supplementary Table S1**) the CTV was the GTV plus a 10 mm safety margin in cranial-caudal direction and a 5 mm safety margin in latero-lateral direction to account for typical anatomical spread (8). The safety margins were manually corrected taking into account anatomical barriers. For cerebral gliomas (patients #2–5, see **Supplementary Table S1**) the CTV was the composite of the FLAIR GTV, the T1-enhancing disease at pre-PT and the surgical cavity plus a 1.5 cm expansion beyond that (9).

### MR Acquisitions

Images were acquired with a 1.5T MR scanner (Philips Ingenia system, Philips Medical Systems, Best, The Netherlands). A 16-channel head coil was utilized, routinely used for brain examinations. MWI was obtained using a multicomponent T2 relaxation technique by a 3D GraSE (10) multi-echo sequence with the following parameters: TR = 1,000 ms, 32 equally spaced echoes ranging from 10 to 320 ms, EPI factor = 5, FOV = 210 × 184 mm^2^, acquisition matrix = 128 × 126 reconstructed to 256x256, 18 contiguous slices 5mm thickness reconstructed to 36 slices 2.5 mm thickness. MWI scans were acquired before PT (pre-PT), at the end of PT (end-PT) and in some cases (see *Results* section) at follow-up. All the patient underwent diagnostic MRI examination before PT and at follow-up, including conventional 3D FLAIR, which was acquired also at the end of PT. FLAIR parameters were TE = 95 ms, TR = 6,000 ms, inversion time = 1,740 ms, contiguous slice thickness = 1.2 mm and pixel size = 0.5 × 0.5 mm^2^ for patient #1 and TE = 150 ms, TR = 4,800 ms, inversion time = 1,660 ms, contiguous slice thickness = 1.15 mm and pixel size = 1.0 × 1.0 mm^2^ for the other patients.

For all patients, MWI and FLAIR pre-PT images were acquired at the beginning of the PT treatment and MWI and FLAIR end-PT images were acquired within one month after treatment. Follow-up diagnostic MR examinations were acquired more than three months after treatment for patients #1–5 and around fifty days after treatment for patient # 6. MWI was also acquired at follow-up for patients #2 and #3 (see below results).

### MWI Post Processing

Multi-echo images were processed on a dedicated workstation by FSL, created by the Analysis Group, FMRIB, Oxford, UK (11), by MatLab (MATLAB^®^, The MathWorks.inc, Natick, MA, USA) and by MERA (12). MERA is a free tool for multi-exponential relaxation analysis, which fits data with a distribution of decaying exponential functions. In the MERA tool (version 2.04) two-hundred logarithmically-spaced time constants, ranging from 1 to 3,000 ms, and a minimum curvature regularization were set. The analysis included fitting the refocusing flip angle (13) as implemented in MERA. Each volume of the multi-echo MRI data was smoothed by a 3D Gaussian kernel (standard deviation equal to 1.5). Multi-echo data were then processed by MERA to identify in each voxel three T2 component from the obtained T2 spectrum. The T2 component below 40 ms was labeled as myelin water (2), the T2 component between 40 and 250 ms was labeled as intra-extracellular water (IEw) and above 250 ms was considered as cerebrospinal fluid/free water. The above 40–250 ms range has been chosen to exclude the myelin water component, but possibly to include tissue alterations (14), hence avoiding the exclusion of potentially informative regions. According to this labeling six 3D maps were extracted, three maps for the weighted average T2 of each compartment and three maps for the relative amplitude of the signal intensity in each compartment. However, the estimated T2 time of the myelin water component is not reliable as only a few echoes go into determining its value. Likewise, the estimated T2 time for CSF is also not accurate, given that the longest measured echo time is only 320 ms. In **Supplementary Figure S1** exemplary images acquired on a healthy volunteer in the implementation phase are shown. In the following sections a specific focus is reported on the analysis of the quantitative IEw T2 map.

### Image Analysis

FLAIR images and IEw T2 maps were aligned by means of FLIRT tools of FSL (15). FLIRT was set with 6 degrees of freedom (i.e. rotations and translations are allowed), trilinear interpolation and using the correlation ratio as the cost function.

The volume of hyper-intensity was manually outlined on FLAIR images on both pre-PT (where it was defined as being the GTV) and end-PT.

On both pre-PT and end-PT data, the volume of hyper-intense IEw T2 inside the CTV was automatically segmented by selecting the voxels with T2 higher than a threshold value. This threshold value was chosen on pre-PT data by the normalized distribution of T2 values, at the intersection between the T2 distribution of the voxels inside the GTV and the T2 distribution obtained on the voxels outside the same GTV. The same threshold value was applied to segment end-PT IEw T2 maps.

A quantitative comparison was performed on both pre- and end-PT between the segmented volumes on T2 maps and the corresponding volumes on FLAIR imaging.

The evolution of the lesion and of the surrounding tissue was visually assessed at pre-PT, at end-PT and at follow-up on the acquired IEw T2 maps and on conventional multi-parameter MRI.

Finally, a quantitative analysis was performed on an identified region of interest (ROI), assessing the corresponding T2 distribution of the ROI at different time points.

## Results

Quantitative volume analysis is reported in **Supplementary Table S2**, where the values of the segmented volumes are reported together with the CTV values. The volume of hyper-intense IEw T2 inside the CTV was segmented by selecting only the voxels with T2 higher than around 88 ms, i.e. the intersection on pre-PT data between the T2 distributions of the voxels inside and outside the GTV (see **Supplementary Figure S2**). The quantitative analysis showed a mismatch at pre-PT examination, i.e. the volume of hyper-intensity in IEw T2 maps exceeded the GTV volume identified on pre-PT FLAIR images. Both the segmented IEw T2 volumes and FLAIR hyper-intensities were greater on pre-PT than on end-PT except for patient #3, where they increased, and for patient #4, where the FLAIR segmented volumes did not change.

Qualitative visual analysis confirmed that for all the patients except patient #4 a spatial mismatch was observed before PT between the IEw T2 quantitative map and FLAIR images, with an area of high T2 that typically extended outside the area identified by abnormal FLAIR hyper-intensity. Only marginal differences were observed in patient #4, with no clear mismatch (not shown). At end-PT, the observed mismatch tends to decrease, by two different evolution patterns.

**Figure 1:**
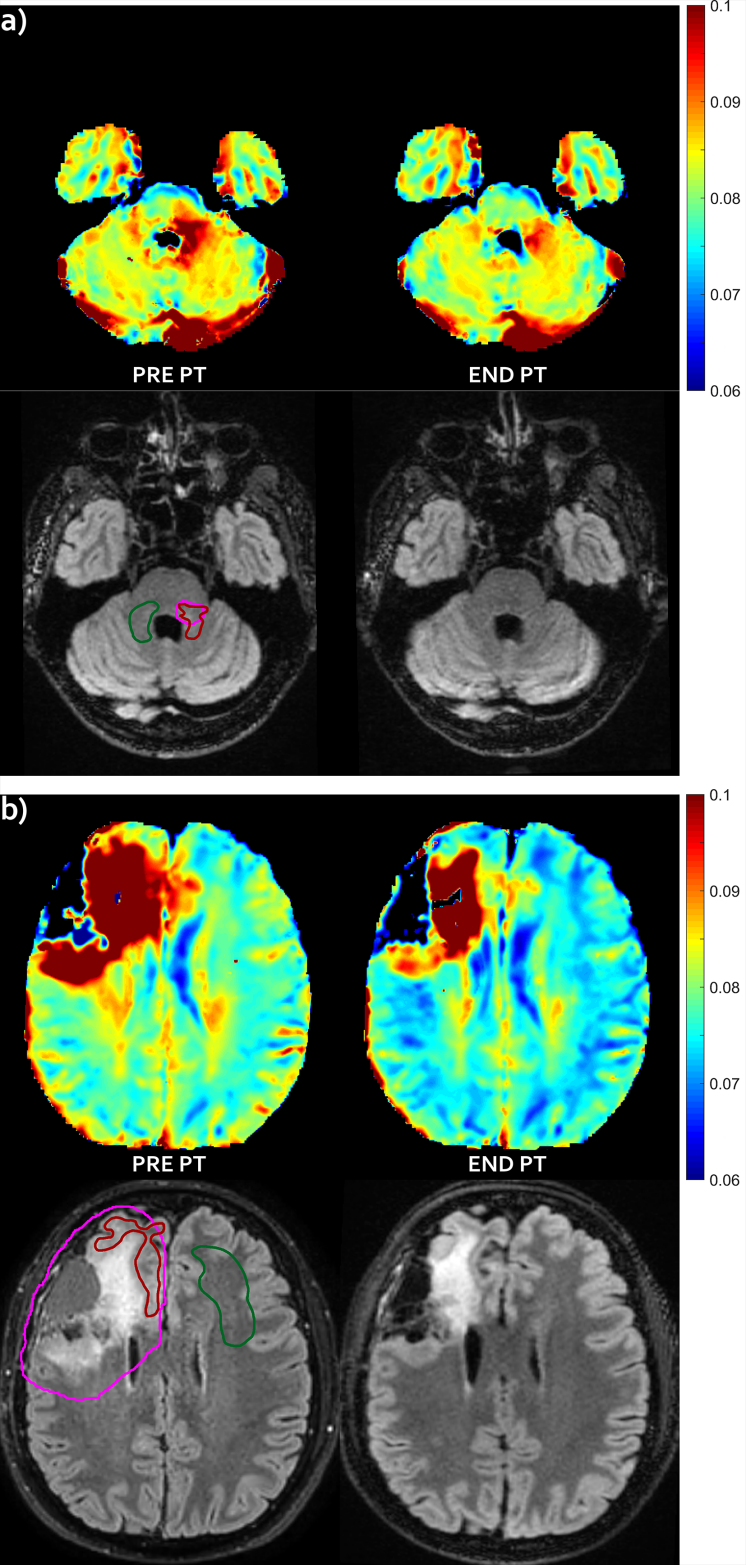
T2 decrease pattern—Evolution pattern observed in patient #1 **(A)** and #5 **(B)**. The mismatch, i.e. abnormal T2 area that extended outside the hyper-intense FLAIR area before proton irradiation (left) markedly reduced at the end of the radiation treatment (right), with no appearance of successive FLAIR hyper-intensity in this region (not shown). For patient #1, no FLAIR abnormality was visible in panel **(A)**, but it was visible at a different slice level (not shown). T2 intra/extracellular water maps (top) and FLAIR images (bottom) are shown. Colored bar is shown in seconds. On pre-PT FLAIR image the contours of the CTV (violet line), of the mismatch ROI (red line) and of the contralateral healthy ROI (green line) are shown.

A reduced extension of the exceeding mismatch region (T2 decrease pattern) was observed for two patients, respectively #1 and #5 (shown in **Figure 1**). Such T2 decrease was confirmed on FLAIR images at follow-up (data not shown).

**Figure 2:**
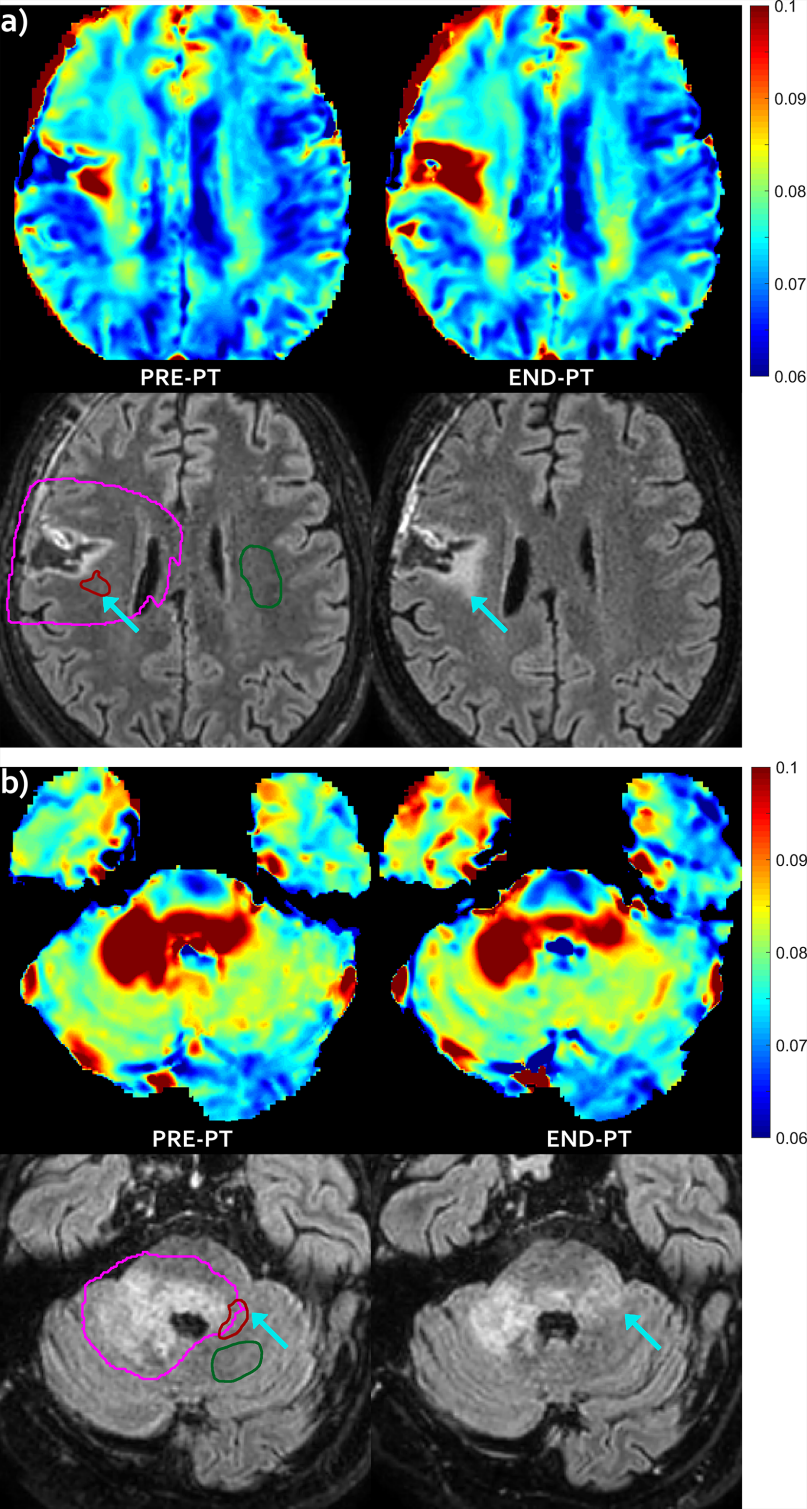
FLAIR increase pattern—Evolution pattern observed in patient #3 **(A)** and in patient #6 **(B)** as highlighted by the arrows. The mismatch, i.e. the abnormal T2 area that extended outside hyper-intense FLAIR area before proton irradiation (left) evolved into a new area of FLAIR hyper-intensity at end-PT (right), that was diagnosed as radiation induced damage. T2 intra/extracellular water maps (top) and FLAIR images (bottom) are shown. Colored bar is shown in seconds. On pre-PT FLAIR image the contours of the CTV (violet line), of the mismatch ROI (red line) and of the contralateral healthy ROI (green line) are shown.

**Figure 3:**
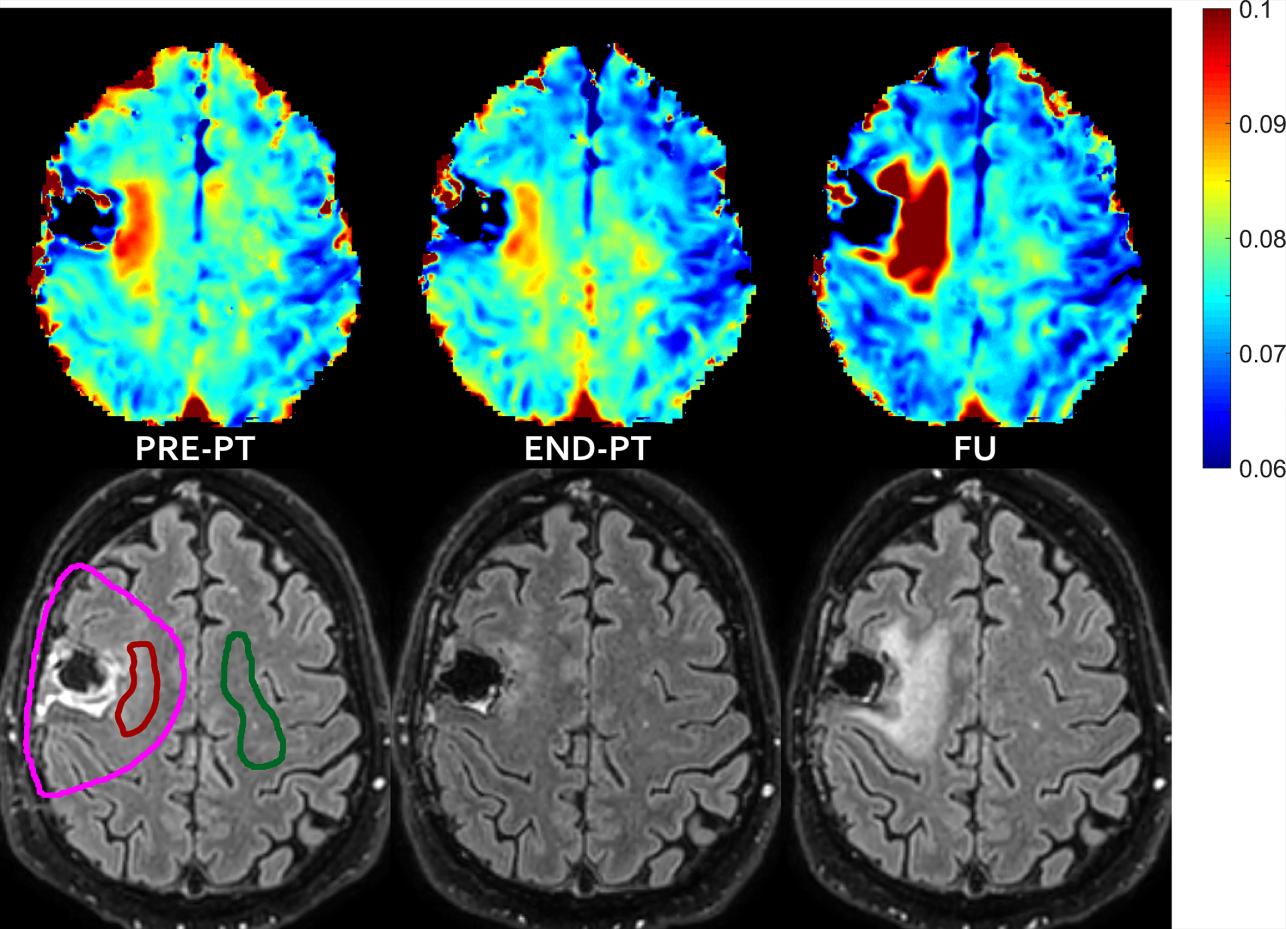
FLAIR increase pattern after temporary T2 decrease—In patient #2 the mismatch, i.e. the abnormal T2 area that extended outside hyper-intense FLAIR area before proton irradiation (left) evolved into new area of FLAIR hyper-intensity diagnosed as radiation induced damage at follow-up (right), with some partial temporary recovery at the end of the radiation treatment (middle). T2 intra/extracellular water maps (top) and FLAIR images (bottom) are shown. Colored bar is shown in seconds. On pre-PT FLAIR image the contours of the CTV (violet line), of the mismatch ROI (red line) and of the contralateral healthy ROI (green line) are shown.

For three patients (#2, #3 and #6) new areas of abnormal hyper-intensity on FLAIR images were observed in the mismatch regions identified before PT (FLAIR increase pattern, which were diagnosed as asymptomatic radiation induced damage at last follow-up examination). Images showing the mismatch on patient #3 and #6 are reported in **Figure 2**. For patient #3 there was a further increase of the hyper-intense area at follow-up (data not shown). For patient #2, the IEw alteration in the mismatch area identified on pre-PT first partially decreased at end-PT and later increased at follow-up, when it was associated with large FLAIR hyper-intensity (**Figure 3**).

**Figure 4:**
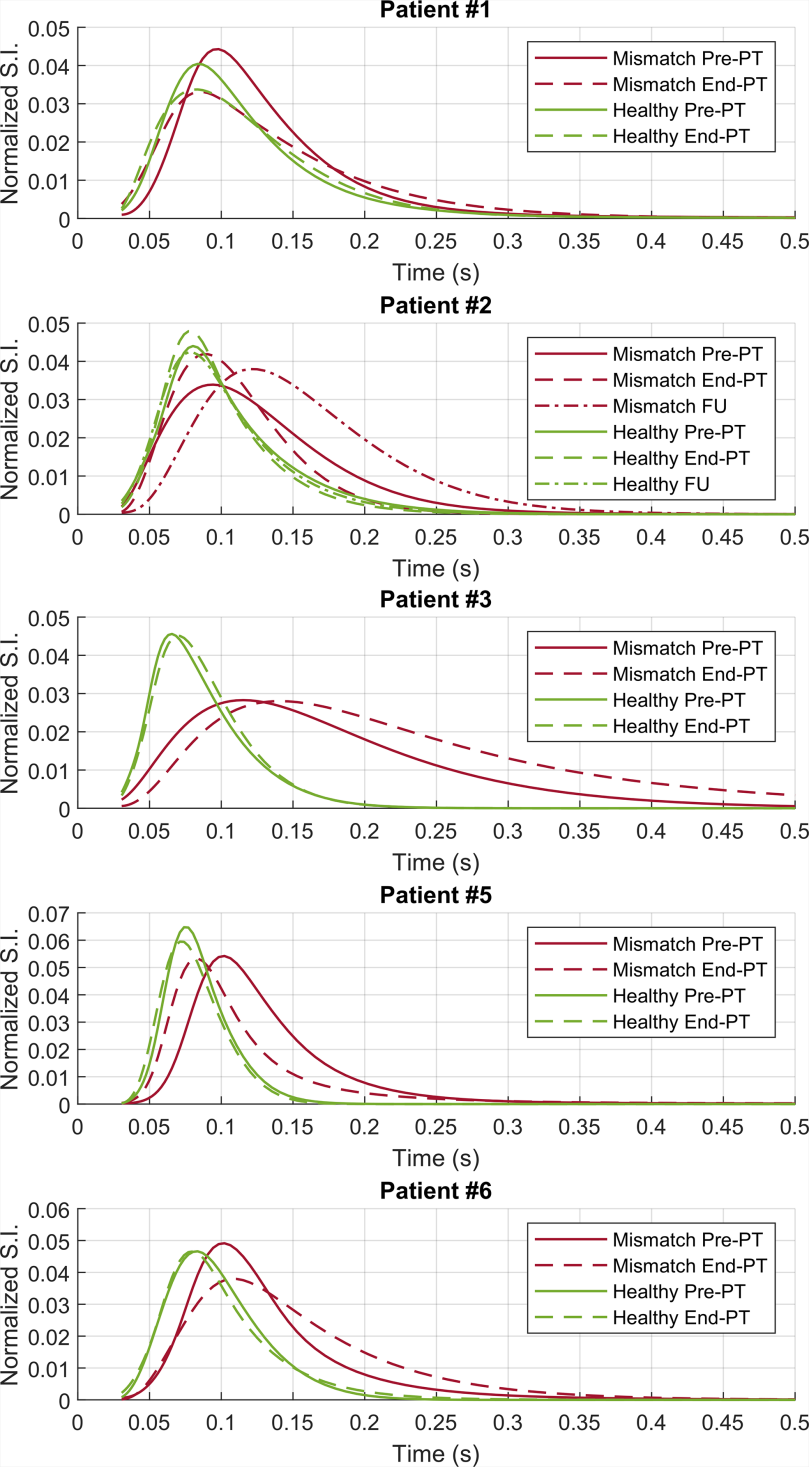
ROI analysis—T2 distributions on the contralateral healthy (green lines) and on the mismatch (red lines) ROIs for patients #1–3 and #5–6. Pre-PT (continuous lines) and end-PT (dashed lines) T2 distributions are shown. For patient #2 also the follow-up T2 distributions (dotted lines) are shown.

A ROI analysis was performed to investigate the characteristics of the T2 distribution that enabled the IEw T2 map to visualize abnormalities not evident with FLAIR imaging (**Figure 4**). For each patient, a mismatch ROI was manually delineated, corresponding to the region where the spatial mismatch was observed before PT between the IEw T2 quantitative map and FLAIR images, as shown in **Figures 1**–**3**. Patient #4 was excluded from the ROI analysis as for this patient it was difficult to identify a mismatch ROI. For comparison, a contralateral ROI on healthy tissue was also manually delineated in the same patients (see **Figures 1** **–** **3**). Whereas the T2 distribution only slightly changed at different time points in the healthy ROIs, it markedly changed in the mismatch ROIs and in a different way among the patients (**Figure 4**). In details, for all the five patients at pre-PT the T2 distribution in the mismatch ROIs showed a shift of the maximum toward higher T2 values and a positive skew of the T2 distribution. Thereafter, at end-PT for patients #1 and #5 it resulted similar to the T2 distribution of the healthy contralateral ROI. These findings were consistent with the observed T2 decrease pattern. On the opposite, at end-PT for patients #3 and #6 there was a further shift and/or a further positive skew of the T2 distribution. These latter findings were consistent with the FLAIR increase pattern observed for these patients. For patient #2 the observed T2 distributions were consistent with a FLAIR increase pattern at follow-up after a temporary T2 decrease at end-PT.

Visual inspection of IEw T2 maps put into evidence a very small decrease in the contralateral healthy tissue after therapy (see **Figures 1B** and **3**). These variations were diffuse in the whole brain, outside the CTV that received full treatment dose. These small changes of few milliseconds were emphasized by the chosen colored map, but they were negligible compared to the changes occurring in the mismatch ROIs, as above clarified in **Figure 4**.

Finally, it was more difficult to clearly identify tissue alterations in the other maps (myelin water maps are exemplarily shown in **Supplementary Figure S3**).

## Discussion

Multicomponent T2 relaxation is one of the MRI methods, generically named by MWI (16–18), developed to investigate demyelinating diseases in the CNS. MWI by T2 relaxation typically provides three main components. While the typical focus of MWI is on the short component arising from water trapped in the lipid bilayer membrane of myelin, our analysis focused instead on the intra/extracellular IEw component and, particularly, on its quantitative map of T2 relaxation times. Indeed, IEw T2 maps describe both the axonal and neural cells and, in our data, showed clear signal alterations.

In this study, we applied for the first time this technique to investigate irradiated brain tumor patients. To our knowledge, only a case report (19) previously described MWI changes after irradiation, by investigating the myelin water fraction without reporting IEw T2 mapping. In that study myelin water maps, which showed some alteration in the white matter, were investigated more than twenty months after radiotherapy, when marked changes were also visible on conventional imaging. Robust and accurate myelin water or myelin lipid imaging might be useful in the early assessment of radiation induced response or in other brain tumor assessment. However, our data did not show any clear alteration of myelin water on visual assessment and thus the analysis was focused on the IEw component.

In particular, a mismatch between IEw T2 maps and FLAIR images was observed in some ROIs of five over six glioma patients, with areas of high T2 that typically extended outside the area identified by abnormal FLAIR hyper-intensity.

In FLAIR imaging, both the myelin water and the fluid component are attenuated, the first due to long echo times with respect to the typical decay time of water trapped in the lipid bilayer, and the latter by an inversion recovery pulse. IEw T2 maps, calculated as the weighted average T2 of the intra/extracellular water, describe a similar compartment and can be somehow considered as a quantitative FLAIR imaging. Due to their quantitative information, IEw T2 maps resulted in a higher sensitivity compared to conventional FLAIR imaging for detecting subtle tissue abnormalities. In fact, the analysis in the mismatch ROIs showed clear differences in the T2 distribution with respect to the healthy tissue at pre-PT. Such differences became even greater for patient #2 at follow-up and for patients #3 and #6 at end-PT. Correspondingly, the mismatch regions resulted clearly hyper-intense also on FLAIR imaging only for patient #2 at follow-up and for patients #3 and #6 at end-PT.

The sequence used in this study is representative of most of the multi-echo sequences used for white matter myelin water studies in the literature and it has been demonstrated to be in agreement with histological measures of myelin content. Since some of the acquisition time is used to sample more echo times instead of the k-space, the improved T2 resolution is obtained at the price of a worse spatial resolution. In this view, it can be considered complementary rather than alternative to FLAIR imaging, which is characterized by poorer T2 resolution (only qualitative) and much better spatial resolution.

Moreover, conventional T2-FLAIR sequence provides the information of both T2 relaxation and proton density of the IEw component, but this study only focused on the T2 relaxation of the IEw component. Indeed, the obtained proton fraction maps were not particularly informative as the subtle alterations, clearly visible on the T2 map, were not visible in the proton fraction map (data not shown). An additional map could be obtained by multiplication of T2 relaxation and the corresponding proton fraction of the IEw. However, this last map does not seem to add too much and it would miss the quantitative information available in the corresponding T2 map.

Since our sampling of the T2 decay curve stopped at a TE of 320 ms, the analysis of longer T2 components was inaccurate. Besides that, using discrete tissue models with a fixed number of components may work quite well in defined systems, such as normal white matter, but for tissue alterations it might be inappropriate, if the model is incorrect. In a previous study (7), T2 components greater than 250 ms were found in a oligodendroglioma and the last TE time of 1,120 ms allowed to measure T2 times of several hundred milliseconds in a glioblastoma. That study by Laule et al. (7) focused on the marked alteration visible on T2-weighted imaging. Accordingly, we also observed some tumor area hyper-intense on FLAIR imaging (data not shown) with a T2 component that was assigned to be a fluid component in our model. However, the focus of the present study was on normal appearing brain regions on T2-weighted FLAIR imaging and, particularly, on the mismatch area identified by IEw T2 mapping. In these regions, the fluid water compartment with high T2 was almost negligible. As evidenced in the T2 distributions obtained by the ROI analysis, the mismatch regions were described by a water compartment whose T2 was in the range of 40–500 ms. The corresponding peak values were approximately in the range 70–140 ms. The echo sampling we used allowed an accurate measurement around those peaks to detect subtle alterations, as small as around 20 ms.

Further and deeper investigations are required to properly assess the specificity of the alteration additionally detected by IEw T2 mapping. MWI is not a well-established widespread used technique and it has to be considered as an experimental method still under development. Visual inspection of IEw T2 maps showed in some cases a diffuse but very small decrease outside the region that received full treatment dose. The ROI analysis demonstrated that these changes in the healthy tissue were negligible compared to the greater changes occurring in the mismatch regions.

The regions characterized by a mismatch between FLAIR and IEw T2 followed two different evolution patterns at follow-up. In the FLAIR increase pattern, tissue alteration might be ascribed to predisposition to tissue damage, which was finally diagnosed as asymptomatic radiation induced damage. Due to initial disease, these areas could have been more sensitive to the radiation insult and might be predictive of the corresponding sequelae.

The T2 decrease seems more difficult to be specified, as the observed mismatch might be due to evolving post-surgical sequelae or oedema but tumor infiltration cannot be excluded a priori. Lower grade gliomas are heterogeneous neoplasms with infiltrative behaviour along white matter tracts and surgical treatment poses special challenge for the neurosurgeon since, due to their diffusely infiltrative growth pattern, intraoperative identification of the exact tumor border is frequently not possible with certainty (20). Lower grade gliomas do not undergo significant gadolinium enhancement and the FLAIR sequences have been shown to precisely demonstrate the margins with increased reproducibility and sensitivity relative to T2 weighted imaging (21). However, morphological MRI tends to underestimate the extent of lower grade gliomas and previous studies using multiple biopsies have identified tumor cells outside the radiological border on FLAIR or T2 weighted sequences (22). In this perspective, the IEw T2 quantitative technique described in our study might be used in pre-operative imaging and correlated with biopsy results.

In conclusion, the contrast obtained in FLAIR imaging is based on the attenuation of the myelin water, due to long echo-times, and of fluid by inversion recovery. A similar attenuation was expected in T2 relaxation when considering only the intra/extracellular water component, thus T2 mapping of the intra/extracellular component can be considered as equivalent to a quantitative FLAIR. In our preliminarily study, T2 mapping of the intra/extracellular component in lower grade gliomas showed a mismatch with FLAIR imaging, with areas of high T2 that typically extended outside the area identified by abnormal FLAIR hyper-intensity. Such mismatch was due to an increased sensitivity of quantitative T2 mapping to detect subtle cerebral tissue abnormalities and deserves to be further investigated in future clinical studies.

## Data Availability Statement

The original contributions presented in the study are included in the article/**Supplementary Materials**, further inquiries can be directed to the corresponding author/s.

## Ethics Statement

The studies involving human participants were reviewed and approved by ethics committee for clinical research of Azienda Provinciale per i Servizi Sanitari (APSS, Trento, Italy). The patients/participants provided their written informed consent to participate in this study.

## Author Contributions

PF and PB contributed to conception and design of the study. PB performed MRI acquisitions and data analysis. PF supported data analysis and data interpretation. UR supported radiological data interpretation. DA, DS, and MA provided patients for the study and supported clinical data interpretation. PF wrote the first draft of the manuscript. PB and UR wrote sections of the manuscript. All authors contributed to the article and approved the submitted version.

## Funding

PB was supported by a grant founded by the Fondazione CARITRO—via Calepina 1, 38122 Trento, Italy.

## Conflict of Interest

The authors declare that the research was conducted in the absence of any commercial or financial relationships that could be construed as a potential conflict of interest.
