# Supplementary material for: Quantitative Multicomponent T2 Relaxation Showed Greater Sensitivity Than Flair Imaging to Detect Subtle Alterations at the Periphery of Lower Grade Gliomas
Source: Front Oncol. 2021 Mar 22;11:651137. doi: 10.3389/fonc.2021.651137 (PMC8019971; doi:10.3389/fonc.2021.651137)
Supplement: Supplementary file 1 [file DataSheet_1.docx]

**Supplementary Table S1. Patient characteristics**

| **Patient**  **No.** | **Age(y)/**  **gender** | **Diagnosis** | **Location** | **Surgery** | **IDH**  **mutation** | **Prescribed dose**  **(Gy RBE)/**  **fractions (n)** | **Time between**  **surgery and MRI**  **pre-PT (days)** |
| --- | --- | --- | --- | --- | --- | --- | --- |
| **1** | 34/M | Grade IV Glioneural tumor | Brainstem | Yes | WT | 54/30 | 51 |
| **2** | 65/M | Grade II glioma | Right frontal | Yes | WT | 59.4/33 | 66 |
| **3** | 54/F | Grade III astrocytoma | Right temporal | Yes | Mut | 60/30 | 106 |
| **4** | 54/F | Grade II astrocytoma | Left frontal | Yes | Mut | 54/27 | 360 |
| **5** | 41/M | Grade III oligodendroglioma | Right frontal | Yes | Mut | 59.4/33 | 113 |
| **6** | 59/M | Diffuse Glioma | Brainstem | No | N.A. | 54/30 | N.A. |

*Abbreviations*: WT = wild type; Mut=Mutation; N.A.= not available.

**Supplementary Table S2. Quantitative volume analysis**

|  | **Pre-PT** | |  | **End-PT** | |  |
| --- | --- | --- | --- | --- | --- | --- |
| **Patient**  **No.** | **IEw T2***  **(cm^3^)** | **FLAIR****  **(cm^3^)** |  | **IEw T2***  **(cm^3^)** | **FLAIR****  **(cm^3^)** | **CTV*****  **(cm^3^)** |
| **1** | 10.1 | 4.1 |  | 3.1 | 2.0 | 18.3 |
| **2** | 14.1 | 3.0 |  | 7.5 | 2.7 | 193.7 |
| **3** | 33.0 | 25.0 |  | 37.3 | 29.0 | 301.4 |
| **4** | 18.6 | 15.8 |  | 14.4 | 16.0 | 174.6 |
| **5** | 87.8 | 25.7 |  | 36.6 | 15.7 | 247.8 |
| **6** | 22.0 | 20.2 |  | 16.9 | 9.5 | 51.0 |

* IEw T2 volumes were segmented by a thresholding procedure;

** FLAIR hyper-intense volumes were manually outlined.

*** CTV = clinical target volume.
