# Supplementary material for: Quantitative Multicomponent T2 Relaxation Showed Greater Sensitivity Than Flair Imaging to Detect Subtle Alterations at the Periphery of Lower Grade Gliomas
Source: Front Oncol. 2021 Mar 22;11:651137. doi: 10.3389/fonc.2021.651137 (PMC8019971; doi:10.3389/fonc.2021.651137)
Supplement: Supplementary file 2 [file DataSheet_2.docx]

**
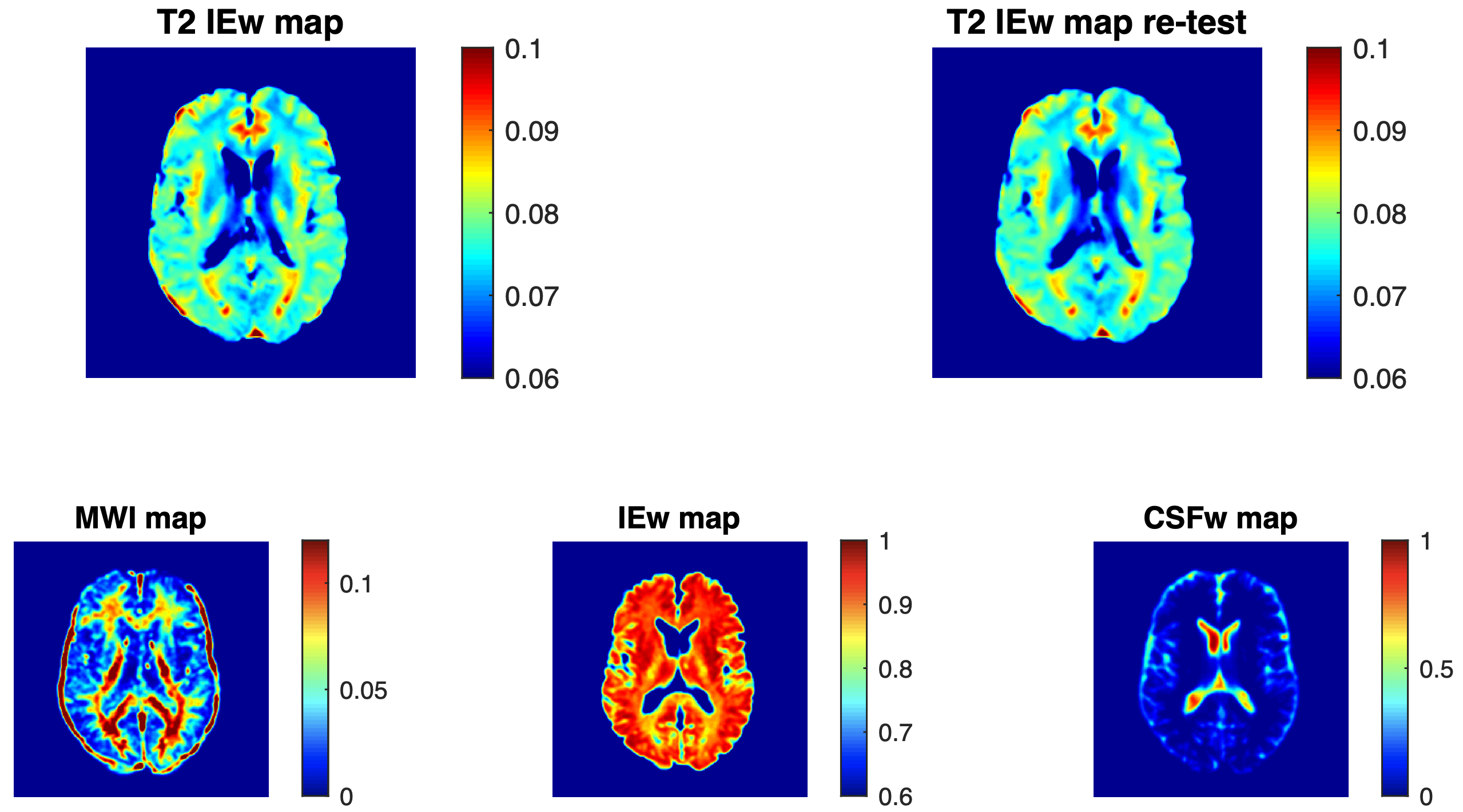
**

**Supplementary Figure S1. Multicomponent T2 relaxation –** Three main compartments were obtained from the T2 spectrum, attributed to water trapped in the lipid bilayer membrane of myelin, intra/extracellular water and a long T2 component, associated to cerebrospinal fluid. The images of a healthy volunteer are shown. The T2 map of the intra/extracellular component is shown in seconds, with colour scale-bar. A retest T2 map, acquired few days later on the same volunteer, is also reported showing accurate reproducibility. In the other maps the relative component is shown. The T2 maps of the myelin and of the fluid component are not shown as they are not reliable due to the echo sampling adopted in the multi-echo sequences.

**
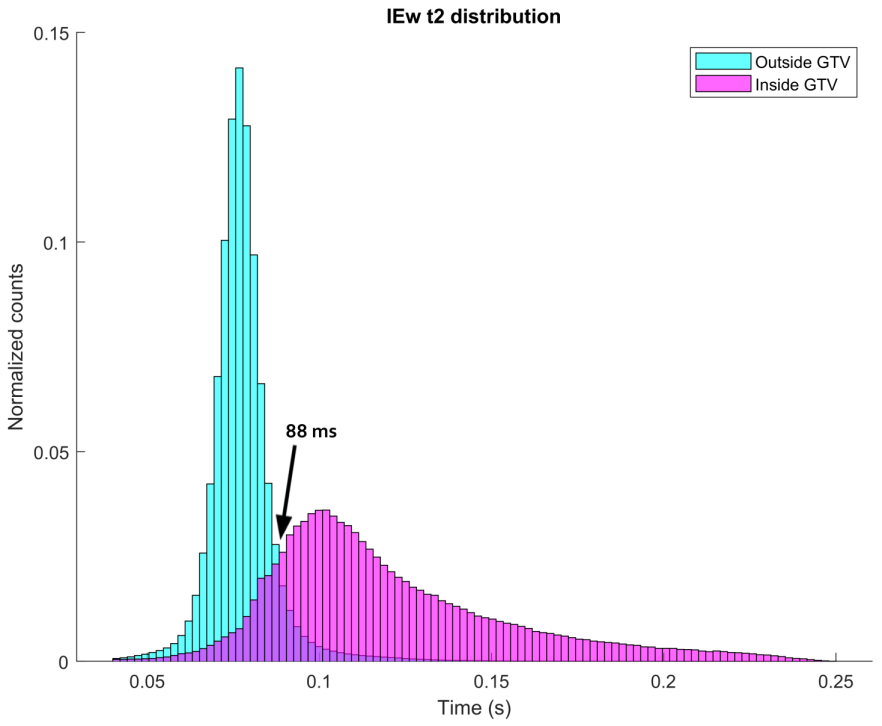
**

**Supplementary Figure S2. Distribution of T2 relaxation times in the intra/extracellular water component (IEw) -** The cumulative IEw T2 distribution (all six patients are plotted together) at pre-PT showed clear differences considering the voxel outside (cyan count-bars) the gross target volume (GTV) and inside the same GTV (magenta count-bars). The T2 value at the intersection between the two distributions (around 88 ms, arrow) was chosen as the threshold to segment the volume of hyper-intense IEw T2 inside the irradiated clinical target volume (CTV) in each patient and at each time point.

**
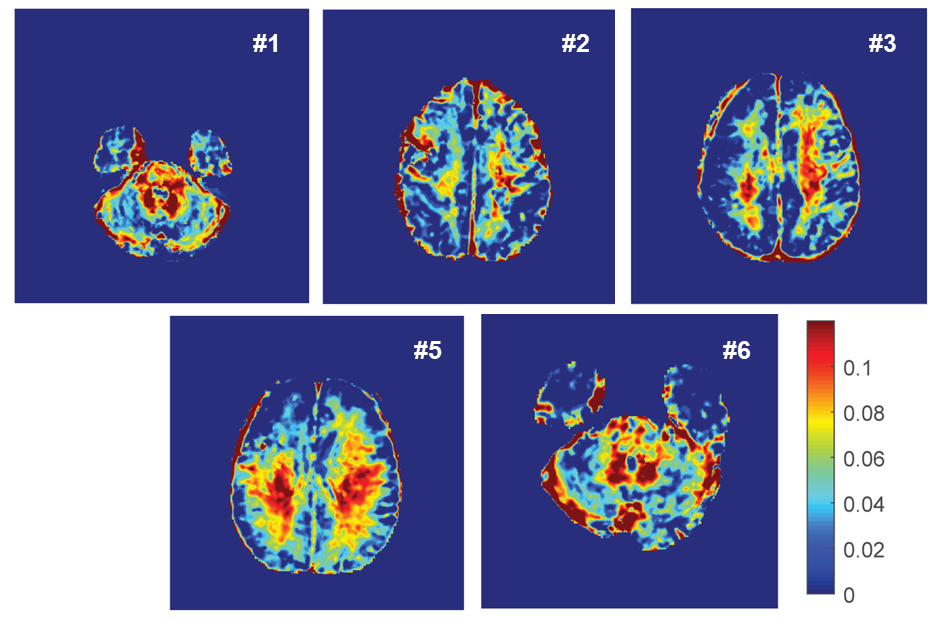
**

**Supplementary Figure S3. Myelin water maps.** Relative component of the myelin water for the five patients who showed a mismatch between the T2 maps of the intra/extracellular water component and the FLAIR images. The reported images were calculated at the same slice level shown in Figures 2-4.
